# Fe_3_O_4_@SiO_2_@Au nanoparticles for MRI-guided chemo/NIR photothermal therapy of cancer cells†

**DOI:** 10.1039/d0ra03699d

**Published:** 2020-07-15

**Authors:** Alexey Maximenko, Joanna Depciuch, Natalia Łopuszyńska, Malgorzata Stec, Żaneta Światkowska-Warkocka, Vadim Bayev, Piotr M. Zieliński, Jaroslaw Baran, Julia Fedotova, Władysław P. Węglarz, Magdalena Parlinska-Wojtan

**Affiliations:** Institute of Nuclear Physics Polish Academy of Sciences Radzikowskiego 152 31-342 Kraków Poland alexey.a.maximenko@gmail.com joannadepciuch@gmail.com; Research Institute for Nuclear Problems of Belarusian State University Bobruyskaya 11 220030 Minsk Belarus; Department of Clinical Immunology, Institute of Pediatrics, Jagiellonian University Medical College Św. Anny 12 Kraków PL-30-663 Poland

## Abstract

Novel functionalized (biofunctionalization followed by cisplatin immobilization) Fe_3_O_4_@SiO_2_@Au nanoparticles (NPs) were designed. The encapsulation of Fe_3_O_4_ cores inside continuous SiO_2_ shells preserves their initial structure and strong magnetic properties, while the shell surface can be decorated by small Au NPs, and then cisplatin (cPt) can be successfully immobilized on their surface. The fabricated NPs exhibit very strong *T*_2_ contrasting properties for magnetic resonance imaging (MRI). The functionalized Fe_3_O_4_@SiO_2_@Au NPs are tested for a potential application in photothermal cancer therapy, which is simulated by irradiation of two colon cancer cell lines (SW480 and SW620) with a laser (*λ* = 808 nm, *W* = 100 mW cm^−2^). It is found that the functionalized NPs possess low toxicity towards cancer cells (∼10–15%), which however could be drastically increased by laser irradiation, leading to a mortality of the cells of ∼43–50%. This increase of the cytotoxic properties of the Fe_3_O_4_@SiO_2_@Au NPs, due to the synergic effect between the presence of cPt plus Au NPs and laser irradiation, makes these NPs perspective agents for potential (MRI)-guided stimulated chemo-photothermal treatment of cancer.

## Abstract 2

Novel functionalized nanoparticles, with toxicity controlled by laser irradiation, are perspective agents for potential (MRI)-guided stimulated chemo-photothermal treatment of cancer.

## Graphical abstract

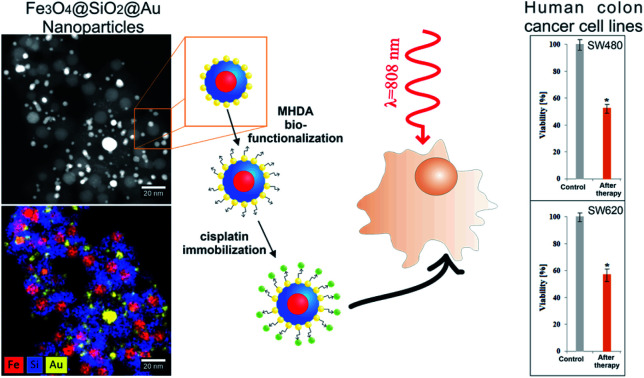

## Introduction

Biocompatible Fe_3_O_4_@Au nanoparticles (NPs), where Fe_3_O_4_ are superparamagnetic cores coated with Au NPs, are building agents with multiple functions for early diagnostics and for new noninvasive therapies for previously incurable diseases. This makes them promising for theranostics, where imaging and therapy modalities are integrated within a single platform.^1,2^ Due to the superparamagnetic features of Fe_3_O_4_ cores, the NPs can be driven in biological objects by the external magnetic field gradient, and can thus be used for labeling of biomolecules and for drug delivery.^3^ Moreover, superparamagnetic Fe_3_O_4_ NPs exhibit strong magnetization in external magnetic field, which contribute to magnetic field perturbations. As a result the dephasing of protons is activated, leading to shortening of the *T*_2_ time (transverse or spin–spin) relaxation of the neighboring regions.^4^ Also superparamagnetic iron oxide NPs with ultrasmall size can influence the *T*_1_ time (longitudinal or spin-lattice) relaxation.^5^ This makes Fe_3_O_4_@Au NPs applicable as contrast agents in MR images. The presence of Au NPs, which have high X-ray absorption, make Fe_3_O_4_@Au NPs also promising for enhancing computed tomography imaging.^6^ On the other hand, superparamagnetic Fe_3_O_4_ NPs are attractive candidates for novel therapeutic approaches for cancer cell selective treatment. Thus, the superparamagnetic Fe_3_O_4_ NPs can be applied in magneto-mechanical cancer treatment, where NPs actuated through the use of super low frequency AC magnetic fields and contribute to cytoskeletal disruption and subsequent cell death, can be selectively enacted upon cancerous cells, while leaving healthy cells intact.^7^ Superparamagnetic Fe_3_O_4_ NPs also can be applied for magnetic particle hyperthermia, where the NPs exposed to alternating magnetic fields of relatively high frequencies (∼100 kHz), generate heat through Néel or Brownian relaxation, which leads to temperature increase, causing subsequent damage to the surrounding cells.^8^

Au NPs demonstrate localized surface plasmon resonance,^9^ based on the collective oscillation of free electrons in conduction bands.^10^ These properties of gold NPs find an application in a minimally invasive technique, which is called photothermal therapy. In this therapy photothermal agents (gold NPs) convert the laser energy to heat, which kills the cancer cells. Moreover, Au NPs are widely used as a platform for drug delivery, for example cisplatin (cPt).^11^ In this case, gold NPs with immobilized cPt provide a synergetic effect in cancer treatment.^12^ And this synergetic effect could also be enhanced in combination with the photothermal therapy described above.^13^

Combination of magnetic iron oxide and gold NPs in the framework of one compact nanoparticle (below 40 nm), in which superparamagnetic properties of iron oxide NPs and surface plasmonic properties of gold NPs complement each other, can noticeably extend the theranostic potential of the system. However, attaching Au NPs to Fe_3_O_4_ is a challenging problem. One of the effective strategy, employs an intermediate adhesion layer of amino-modified silica, which coats the Fe_3_O_4_ NPs, and to which Au NPs are attached.^14,15^ The latter strategy allows to use hydrophobic iron oxide NPs prepared through thermal decomposition in non-aqueous media, which are nearly monodispersive.^16,17^ Silica shells also can effectively enhance biocompatibility, prevent corrosion and agglomeration of Fe_3_O_4_ NPs and improve their stability in aqueous solution. The presence of silanol functional groups on the surface of the core–shell Fe_3_O_4_@SiO_2_ NPs makes them dispersible in polar solvents such as water, and also provides easy further modification with other functional groups, thus enabling coupling with different drugs and biomolecules. The immobilization of cPt on the nanoparticles surface, can increase the therapeutic influence of the drug and make its impact more controllable. However, the combination all of the three components in one system is a challenging problem.

**Fig. 1:**
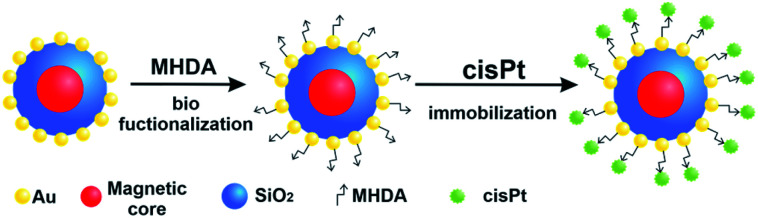
Scheme of the Fe_3_O_4_@SiO_2_@Au NPs biofunctionalization process and process of the cPt immobilization.

Here we design novel, targeted cPt-based Fe_3_O_4_@SiO_2_@Au NPs (Fig. 1), which will have an improved potential in magnetic resonance imaging (MRI)-guided chemo-photothermal stimulated treatment of cancer. For this purpose, our magnetic NPs were functionalized by cPt, a metallic platinum coordination compound with a square planar geometry, which shows high anticancer activity in a variety of tumors, and which is widely used in chemotherapy medication to treat a number of cancers.^18^ For the theranostic application, we tested the effectiveness of the NPs as contrast agents for MRI and investigated *in vitro* the chemo-phototherapy potential of the Fe_3_O_4_@SiO_2_@Au nanosystem on two colon cell lines: SW480 and SW620 in terms of cell viability (MTT assay) and morphological changes induced in tumor cells (optical microscopy). Because near-infrared (NIR) radiation can penetrate into biological tissues several centimeters deep, we chose a laser with an 808 nm wavelength, which is employed in photothermal therapy in the majority of cases.^19^ The morphology, structure and magnetic properties of the Fe_3_O_4_@SiO_2_ and Fe_3_O_4_@SiO_2_@Au nanosystems have been investigated using transmission electron microscopy (TEM), X-ray diffraction (XRD), Mössbauer spectroscopy and SQUID-magnetometry. The stability of the functionalized Fe_3_O_4_@SiO_2_@Au NPs and the verification of the effective process of biofunctionalization and cPt immobilization have been carried out using differential scanning calorimetry (DSC), thermogravimetric analysis (TGA) and Raman spectroscopy.

## Experimental section

### Materials

Tetraethyl *ortho*-silicate (TEOS, 99.0%), (3-aminopropyl)triethoxysilane (APTES, 99%), polyoxyethylene (5) nonylphenylether (Igepal CO-520), tetrakis(hydroxymethyl)phosphonium chloride (THPC, 80% solution in H_2_O), hydrogen tetrachloroaurate(iii) hydrate (HAuCl_4_·3H_2_O, 99.9% metals basis), 16-mercaptohexadecanoic acid (16-MHDA), dimethylformamide (DMF), pentafluorophenyl (PFP), *N*,*N*-diisopropylethylamine (DIPEA) and *N*-cyclohexyl-*N*-(2-morpholinoethyl)carbodiimide metho-*p*-toluenesulfonate (CMC) were purchased from Sigma Aldrich (USA). Ferric chloride hexahydrate (FeCl_3_·6H_2_O, 99%), sodium oleate (NaOA, 95%) were purchased from Carl Roth. 1-Octadecene (ODE, tech. 90%), oleic acid (OA, tech. 90%), potassium carbonate (K_2_CO_3_, 99.0%) were purchased from Alfa-Aesar Chemicals. Cyclohexane (C_6_H_12_, 99.5%), hexane (C_6_H_12_, 97.0%), acetone (C_3_H_6_O, 99.5%), ammonia solution (NH_3_·H_2_O, 25%), chloroform (CHCl_3_), ethanol (C_2_H_5_OH, 96%) were bought from POCH. Ultrapure water was used throughout the experiments. All the chemicals were used as received without further purification.

### Synthesis of Fe_3_O_4_@SiO_2_@Au NPs

The process of synthesis involved three stages: (a) the synthesis of hydrophobic Fe_3_O_4_ NPs, (b) encapsulation of the Fe_3_O_4_ NPs in an SiO_2_ shell and (c) attaching the Au NPs to the Fe_3_O_4_@SiO_2_.

#### (a) Hydrophobic Fe_3_O_4_ NPs

Hydrophobic Fe_3_O_4_ NPs were fabricated according to the 2-step thermal-decomposition method explained elsewhere.^16,17^ Shortly, in first step an iron-oleate complex was prepared. For this purpose, a solution of iron chloride (FeCl_3_·6H_2_O, 3.6 g) and sodium oleate (12.2 g) dissolved in a mixture of ethanol (27 mL), hexane (47 mL) and distilled water (20 mL) in a 250 mL necked round bottom flask was prepared. The above-mentioned solution was heated to 70 °C with a constant heating rate of 3.3 °C min^−1^ under Ar flow and was kept at that temperature for 4 hours. After the reaction was complete the upper red brown layer of iron oleate was washed three times with distilled water in a separatory funnel and subsequently dried in air. In the second step, the synthesized iron-oleate complex (9 g) with oleic acid (1.43 g) were dissolved in 1-octadecene (63 mL) at room temperature. The solution placed in a 250 mL necked round bottom flask was heated with a constant heating rate of 3.3 °C min^−1^ to reflux (318 °C) under argon for 30 min. At the end of the reaction the brownish-black solution was cooled to room temperature and the obtained NPs were precipitated in ethanol (200 mL) and centrifuged for 5 minutes in 50 mL flacons at 4000 rpm. Then the NPs were re-dispersed in chloroform and isolated by adding acetone and centrifuging the obtained solution. This washing step was repeated two times and the third time the NPs were left in a chloroform/acetone mixture for 24 hours. The NPs were subsequently collected by centrifugation, dried, re-dispersed and stored in cyclohexane, where the concentration of NPs was set to 2.5 g L^−1^.

#### (b) Fe_3_O_4_@SiO_2_ NPs

The Fe_3_O_4_@SiO_2_ complexes were fabricated by reverse microemulsion method.^20^ Briefly, Igepal CO-520 (1.0 g) was dispersed in cyclohexane (22 mL) and sonicated for 10 min. Then, 25%-ammonium hydroxide (0.4 mL) was added to 1 mL of the as-prepared Fe_3_O_4_ NPs cyclohexane solution and stirred for 30 min. Subsequently, TEOS (0.07 mL) was added to the above mixture and the final solution was left under continuous stirring for 12 hours. After this, APTES (0.05 mL) was dropped into the mixture, which was left under continuous stirring for another 12 hours. The obtained Fe_3_O_4_@SiO_2_ core–shell NPs were collected by centrifugation and washed with ethanol three times and re-dispersed in 1 mL of ethanol.

#### (c) Fe_3_O_4_@SiO_2_@Au NPs

The procedure of Fe_3_O_4_@SiO_2_ NPs decoration by Au NPs can be divided into two steps. The first step is the synthesis of Au NPs and the second step is attaching the Au NPs on the surface of Fe_3_O_4_@SiO_2_, which is functionalized with APTES. Au NPs with diameters from 2 nm to 6 nm were synthesized by direct reduction of HAuCl_4_·3H_2_O with THPC previously reported in the literature.^21^ Briefly, portions of sodium hydroxide (1 mol, 0.5 mL), the reducing agent THPC (1% aqueous solution, 1 mL), and the metal salt HAuCl_4_ (1% aqueous solution, 2 mL) were added successively to 38 mL of water while stirring and the resulting solution was aged for a few days at 4 °C, till orange-brown hydrosols of gold were formed. Then the solution of Fe_3_O_4_@SiO_2_ NPs (0.5 mL) was mixed with the 5 mL of gold seed solution. The obtained Fe_3_O_4_@SiO_2_@Au NPs were collected by magnet and washed with water and ethanol for two times.

### Biofunctionalization of the Fe_3_O_4_@SiO_2_@Au NPs and immobilization of cPt on their surface

In order to attach the drugs to the stabilized Fe_3_O_4_@SiO_2_@Au NPs, 16-MHDA was used as a linker. This chemical compound has 16 carbon atoms, a thiol group (–SH) on one side and a carboxyl group on the other side. Thanks to the thiol group, the 16-MHDA is linked with the NPs surface and the COOH group is linked with cPt. Moreover, using this linker, the active surface of Au NPs was not coated, as it is in the case when *e.g.* polyethylene glycol (PEG) shell was used. First, the NPs were incubated with MHDA for overnight at 4 °C. After rinsing with DMF, the MHDA-covered NPs-structures were incubated in DMF solution of PFP, DIPEA and CMC, during 30 min at 25 °C. After repeated rinsing with DMF and centrifugation, a cPt solution was added and incubated for 30 min at 25 °C. The scheme of functionalization (biofunctionalization and immobilization of cPt on their surface) of Fe_3_O_4_@SiO_2_@Au NPs is shown in Fig. 1.

### TEM characterization

The morphology of the fabricated NPs was investigated by scanning transmission electron microscopy (STEM) using the high-angle annular dark-field detector (HAADF). Selected area electron diffraction (SAED) patterns were taken in the TEM mode. Energy dispersive X-ray spectroscopy (EDS) was used to analyze the chemical composition of the synthesized NPs. All these measurements were performed on an aberration-corrected FEI Titan electron microscope operating at 300 kV equipped with a FEG cathode. The particle size distribution was evaluated based on the HRSTEM images taken from different areas of the TEM grids.

### X-ray diffraction

The crystal microstructure of the fabricated NPs was analyzed using a two-circle laboratory diffractometer Panalytical X'Pert Pro. The measurements were performed in the standard *θ*–2*θ* geometry using Cu K_α_ radiation (*λ* = 0.15406 nm). The dispersion of the NPs was dried on a zero-background holder and was placed on a sample spinner with rotation time adjusted to 16 seconds. The data were collected in the range between 25–85° (2*θ*) with a step size of 0.08°.

### Mössbauer spectroscopy

Mössbauer spectra were recorded in transmission geometry at room temperature and at 16 K using the spectrometer MS4 (SeeCo, USA) with a ^57^Co/Rh source (12 mCi). Low temperature measurements were performed using a closed cycle refrigerator system CCS-850 (Janis Research Company, USA). The temperature was controlled using a Lakeshore temperature controller LS335 (Lake Shore Cryotronics, Inc., USA) with dual calibrated DT-670 sensors with an accuracy of ±0.005 K. The spectra were fitted using the MOSMOD software, assuming a Gaussian distribution of the hyperfine magnetic field (*H*_hf_) and quadrupole splitting within the iron nuclei.^22^ Lorentzian line shape of source natural line width was determined from the Mössbauer spectrum of a pure 28 μm thick α-Fe foil. The spectrometer was calibrated to pure α-Fe by collecting the spectra of a standard α-Fe foil at room temperature and 16 K. All isomer shifts (IS) are presented in respect to an α-Fe standard.

### Magnetometry

A SQUID-magnetometer (MPMS XL, Quantum Design) was used to investigate the magnetic properties of iron-oxide NPs. The zero-field cooled (ZFC) temperature dependences of the magnetization were measured by cooling the sample from 300 K to 5 K and then applying a field of 100 Oe to record the magnetization, while the sample was heated from 5 K to 300 K. The field cooled (FC) measurements were made with a field of 100 Oe applied during the cooling process. The hysteresis curves were measured at 5 K and 300 K, respectively.

### UV-Vis measurements

The UV-Vis measurements were performed with a Lambda Bio20 instrument from PerkinElmer. The resolution was chosen to be 1 nm and the scan speed was 240 nm min^−1^. The spectral range was from 200 nm to 800 nm.

### MRI

To investigate the effectiveness of the NPs as contrast agents, MR imaging as well as relaxometry were performed using a 9.4T Bruker Biospec 94/20 MRI scanner. For the acquisition of the series of axial images of each sample, the RARE with variable repetition time *T*_R_ (RAREVTR) and the MSME imaging sequences were used. Application of RAREVTR sequence allows to obtain simultaneously *T*_1_ and *T*_2_ relaxation times maps of samples with different concentrations of the NPs. MSME sequence was employed in order to increase the number of echoes and therefore to obtain better quality of *T*_2_ measured and *T*_2_-weighted images. For acquired *T*_1_ and *T*_2_ values, linear regression of relaxation rates: *R*_1_ = *T*_1_^−1^ and *R*_2_ = *T*_2_^−1^*vs.* NPs concentration in samples was performed:

1 *R*_1,2_ = *R*_1S,2S_ + *r*_1,2_*C*

where: *C* – the concentration of NPs, *R*_1,2_ the relaxation rate of a whole sample, *R*_1S,2S_ – the relaxation rate of a solvent without contrasting agent, *r*_1,2_ – the relaxivity of Fe_3_O_4_ NPs.

### FTIR measurements

The Fourier transform infrared absorption (FT-IR) spectra in the wavelength range between 400–4000 cm^−1^ were acquired using an EXCALIBUR FTS-3000 spectrometer at room temperature and measured for the respective samples mixed with KBr. The sample was dried and sandwiched between two KRS-5 window disks. The 64 scans were averaged at a resolution of 4 cm^−1^. During the experiments, the spectrometer was purged with dry nitrogen. Baseline correction and normalization of FTIR spectra were applied.

### Calorimetric and TGA measurements

Calorimetric measurements were carried out under dry 5.0 pure nitrogen purge (25 mL min^−1^) on a TA Instruments' DSC 2500 differential scanning calorimeter equipped with a liquid nitrogen LN2P cooling pump. Thermal behaviour of samples placed in aluminium pans and crimped with hermetic lids, was studied in a temperature range from 5 °C to 250 °C with a maximum heating rate of 5 °C min^−1^. Calibrations of the temperature and enthalpy were performed using an indium standard. Additionally, modulated differential scanning calorimetry was employed to gain more thorough insight into thermal properties of the specimens. TGA experiments were carried out under flowing synthetic air (N_5.0_) on a TA Instruments' TGA 5500 high-resolution thermogravimetric analyser using platinum 100 μL pans with a maximum heating rate of 5 °C min^−1^. The specimens were either directly placed in open 100 μL platinum pans or previously enclosed inside aluminium pans to limit the evaporation rate and measured in a temperature range from the ambient temperature to 500 °C. The temperature calibration was carried out using nickel and alumel standards. Additionally, high resolution (HiRes TGA) mode was employed to gain more precise values of the experimental points.

### FT-Raman spectroscopy

FT-Raman spectra were recorded using a Nicolet NXR 9650 FT-Raman Spectrometer equipped with an Nd:YAG laser (1064 nm) and a germanium detector. The measurements were performed in the range from 150 cm^−1^ to 3.700 cm^−1^ with a laser power of 1 W. The unfocused laser beam was used with a diameter of approximately 100 μm and a spectral resolution of 8 cm^−1^. Raman spectra were processed by the Omnic/Thermo Scientific software based on 128 scans.

### Cell lines

As the *in vitro* model, two human colon cancer cell lines (SW480 and SW620) were used, which were obtained due to the courtesy of Prof. Caroline Dive, Paterson Institute for Cancer Research, University of Manchester. These cell lines were cultured in DMEM with high glucose (Corning, NY, USA) in a 37 °C humidified atmosphere with 5% CO_2_. All media were supplemented with 10% fetal bovine serum (FBS, Biowest, Nuaille, France) and gentamicin (50 μg mL^−1^), (PAN-Biotech, Aidenbach, Germany). The cells were cultured by bi-weekly passages and were regularly tested for *Mycoplasma* sp. contamination by PCR-ELISA kit (Roche, Mannheim, Germany) according to the manufacturers' instruction.

### Light microscopy images of cells

The images of cells at 100× magnification were taken using Microscope Olympus IX70 (Olympus Corporation, Tokyo, Japan).

### Light source and cells irradiation protocols

The irradiation of the cells was conducted by a low-intensity laser operating at 808 nm wavelength. An adjustable power supply was connected to the setup to enable control of the power output of the laser with intensity of 100 mW cm^−2^ and irradiation time of 5 minutes. The description of the all samples, which were studied in this experiment, is presented in Table 1.

**Table 1:** Description of samples

Sample	Name of the samples in the manuscript
Control samples of SW480 and SW620 cell lines (cells without addition of other substances and without laser irradiation)	Ctrl
Cells cultured with Fe_3_O_4_@SiO_2_@Au NPs	C@NPs
Cells cultured with 5 μM of cisplatin	C@cPt
Cells cultured with functionalized Fe_3_O_4_@SiO_2_@Au NPs	C@cPtNPs
Control samples irradiated by the 808 nm laser	C@808
Cells cultured with Fe_3_O_4_@SiO_2_@Au NPs and irradiated by the 808 nm laser	C@808NPs
Cells cultured with 5 μM cisplatin and irradiated by the 808 nm laser	C@808cPt
Cells cultured with functionalized Fe_3_O_4_@SiO_2_@Au NPs and irradiated by the 808 nm laser	C@808cPtNPs

### MTS assay

Cytotoxic activity of NPs, cPt, laser irradiation and combination of these three factors against human colon cancer cells (SW480 and SW620) was determined using 3-(4,5-dimethylthiazol-2-yl)-5-(3-carboxymethoxyphenyl)-2-(4-sulfophenyl)-2*H*-tetrazolium (MTS) assay (CellTiter 96® AQueous One Solution Cell Proliferation Assay, Promega, Madison, WI). Briefly, the cells were cultured in flat-bottom 96-well plates (Sarstedt, Numbrecht, Germany) at a density 1 × 10^4^ per well in DMEM medium containing 10% FBS. After 24 h, 20 μL of 50 μg mL^−1^ functionalized Fe_3_O_4_@SiO_2_@Au NPs as well as non-functionalized solutions were added to the cells. After additional 24 hours of culture, 20 μL of MTS (CellTiter 96® AQueous One Solution Cell Proliferation Assay, Promega) dye solution was added per well and incubated for 1.5 h. The quantity of formazan product, directly proportional to the number of living cells in culture, was detected by absorbance measurements at 490 nm with a 96-well plate reader (Spark® Tecan, Mannedorf, Switzerland).

### Analysis of cell viability data

The obtained MTS assay results are represented as the means ± SEM (the standard error of the mean). The quantitative results were finally compared with the *T* test. *P* value < 0.05 was considered to be statistically significant. Moreover, in the MTS test, data for each treatment point shows an average from three parallel wells. The data were analyzed and presented graphically using the Origin Lab2019b software.

## Results and discussion

### Characterization of nanoparticles

**Fig. 2:**
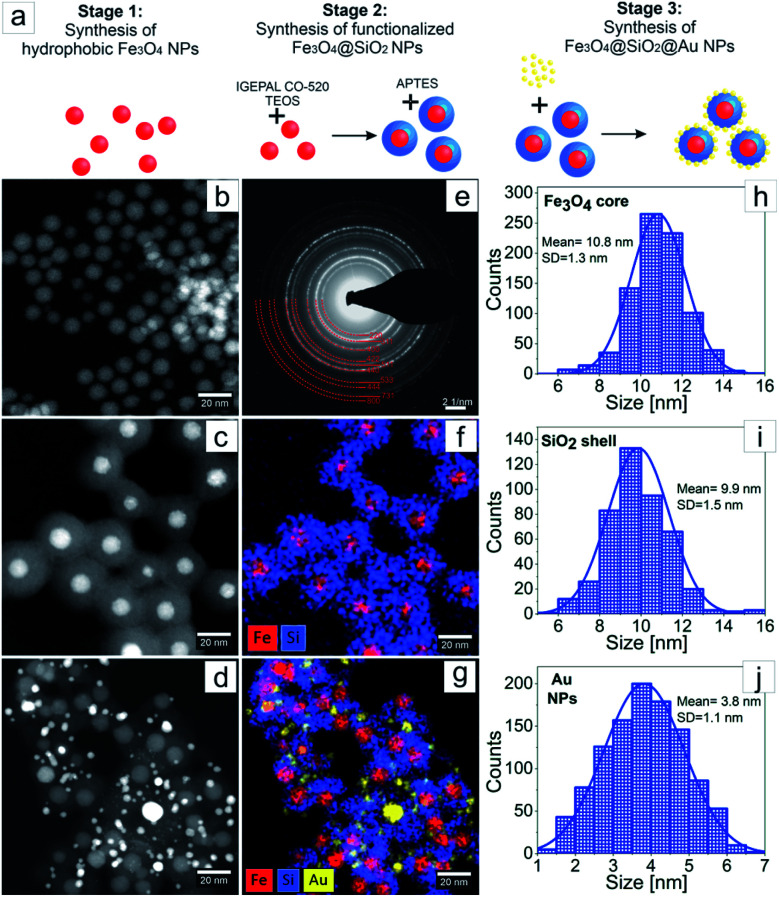
Schematic diagram of the three stages of Fe_3_O_4_@SiO_2_@Au NPs synthesis (a); HAADF STEM overview of Fe_3_O_4_ (b), Fe_3_O_4_@SiO_2_ (c) and Fe_3_O_4_@SiO_2_@Au (d) NPs; SAED pattern of Fe_3_O_4_ NPs (e); EDX elemental maps of Fe, Si, and Au distribution in Fe_3_O_4_@SiO_2_ (f) and Fe_3_O_4_@SiO_2_@Au (g) NPs; the distributions of: Fe_3_O_4_ NPs size (h), SiO_2_ shell thickness (i) and Au NPs size (j).

The three stages of Fe_3_O_4_@SiO_2_@Au NPs synthesis are schematically represented in the Fig. 2a. In the first stage, synthesis of hydrophobic monodisperse spherical Fe_3_O_4_ NPs was made by thermal decomposition of iron precursor using oleic acid as surfactant.^16,17^ The purpose of the second stage was encapsulating of the Fe_3_O_4_ NPs by a full SiO_2_ shell by reverse microemulsion method.^20^ At this stage two subsequent ligand exchange reactions occurred on the surface of the Fe_3_O_4_ NPs. At first, the ligand exchange was between chemically adsorbed oleic acid and surfactant Igepal CO-520, and as a result Igepal CO-520 was chemically adsorbed on the Fe_3_O_4_ NPs surface. Then, TEOS was added to the mixture where it hydrolyzed, performed ligand exchange with Igepal CO-520 and chemically adsorbed on the Fe_3_O_4_ NPs surface. The adsorbed TEOS forms an SiO_2_ shell after its condensation process. At the end of the second stage, the surface of the core–shell NPs was functionalized with amino groups using APTES, where the zeta potential value of the NPs surface was increased to 40 mV allowing thus for sequential adsorption of Au NPs on the surface of Fe_3_O_4_@SiO_2_ NPs was allowed.^15^ At the third stage Au NPs were fabricated by direct reduction method, where HAuCl_4_·3H_2_O was used as gold precursor and tetrakis (hydroxymethyl)phosphonium chloride (THPC) as reducing agent.^21,23^ The fabricated Au NPs were mixed with the solution of functionalized Fe_3_O_4_@SiO_2_ and adsorbed on their surface. As a result, Fe_3_O_4_@SiO_2_@Au NPs were obtained. The morphology investigation and chemical characterization of the NPs at every stage of the synthesis were made by transmission electron microscopy (Fig. 2). In Fig. 2b–d we present STEM HAADF images of Fe_3_O_4_, Fe_3_O_4_@SiO_2_ and Fe_3_O_4_@SiO_2_@Au NPs, respectively. In Fig. 2e, the SAED pattern for the Fe_3_O_4_ NPs after synthesis is shown. The EDX maps of Fe_3_O_4_@SiO_2_ NPs and Fe_3_O_4_@SiO_2_@Au NPs are presented in Fig. 2f and g. Moreover, the distributions of Fe_3_O_4_ NPs size, SiO_2_ shell thickness and Au NPs size obtained from the analysis of STEM HAADF images can be found in Fig. 2h–j. As it can be seen from Fig. 2b and h in the first stage of synthesis, monodispersed Fe_3_O_4_ NPs with the average diameter of 11 nm were fabricated. The diffraction rings on the SAED pattern, Fig. 2e, were indexed with the planes corresponding to inverse spinel crystal structure typical for Fe_3_O_4_.^23^ The successful and uniform encapsulation of Fe_3_O_4_ NPs by the SiO_2_ shell in the second stage of the synthesis is presented in Fig. 2c and f, where a well-defined core–shell structure with one iron-oxide core surrounded by a homogeneous SiO_2_ shell is observed. The average thickness of the shell is around 10 nm (Fig. 2i). In the third stage the Fe_3_O_4_@SiO_2_ core–shell NPs were decorated with Au NPs with an average size of 4 nm, Fig. 2d, g and j. From Fig. 2d and g, it can be seen that every Fe_3_O_4_@SiO_2_ core–shell nanoparticle has a few Au NPs on its surface.

**Fig. 3:**
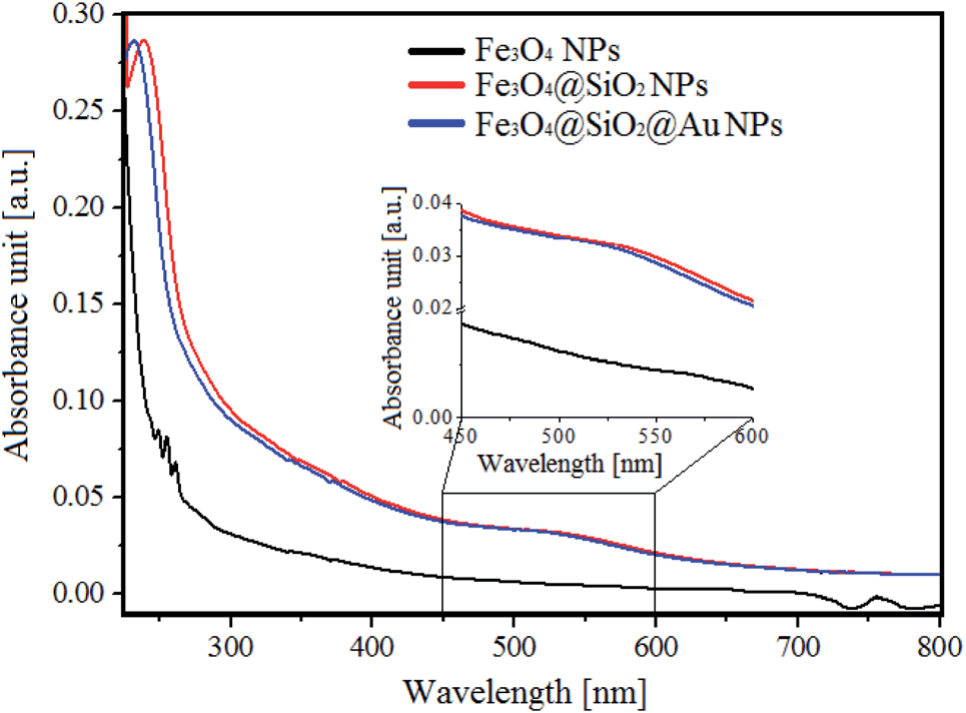
UV-Vis spectra of: Fe_3_O_4_ NPs (black colour), Fe_3_O_4_@SiO_2_ NPs (red colour) and Fe_3_O_4_@SiO_2_@Au (blue colour).

The UV-Vis absorption spectra of different Fe_3_O_4_ NPs with and without a SiO_2_ shell as well as Fe_3_O_4_@SiO_2_ decorated by Au NPs are presented in Fig. 3. In the UV-Vis spectrum of pure Fe_3_O_4_ NPs, no obvious peaks were observed. However, encapsulation of the magnetic NPs in a 10 nm SiO_2_ shell, caused the appearance of a peak at 250 nm in the spectra of the Fe_3_O_4_@SiO_2_ NPs. This peak corresponds to the created core–shell structure of the NPs, and may originate from the changes of the band gap caused by the quantum size effect and surface effect of nanostructures,^24^ as well as from the Fe–O–Si bonds of the core–shell NPs.^25^ Moreover, decorated Fe_3_O_4_@SiO_2_ NPs with 4 nm Au NPs caused the appearance of a broad peak around ∼540 nm, which originates from the gold NPs.^26^ Interestingly, the peak at 250 nm, which was visible in the UV-Vis spectrum of Fe_3_O_4_@SiO_2_, was shifted towards lower wavelengths. This can suggest, that the synthesis of Au NPs on the Fe_3_O_4_@SiO_2_ NPs surface caused changes in the Fe–O–Si bonds, which are, as most number of chemical bonds, sensitive for the local environmental changes.^25^

**Fig. 4:**
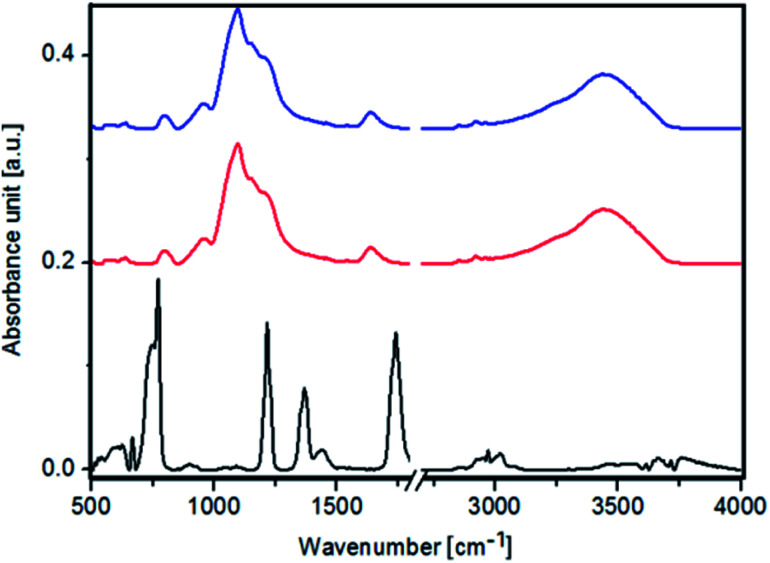
FTIR spectra of: Fe_3_O_4_ NPs (black color), Fe_3_O_4_@SiO_2_ NPs (red color) and Fe_3_O_4_@SiO_2_@Au (blue color).

In the FTIR spectra of pure Fe_3_O_4_ NPs, Fe_3_O_4_@SiO_2_ NPs and Fe_3_O_4_@SiO_2_@Au (Fig. 4) a peak at 590 cm^−1^ corresponding to Fe_3_O_4_ is observed. Moreover, peaks at 1110 cm^−1^, 920 cm^−1^, 790 cm^−1^, 670 cm^−1^ originating from FeOH are noticed. Furthermore, peaks characteristic for γ-FeO(OH) and α-FeO(OH) are visible in FTIR spectra of Fe_3_O_4_ NPs at 1026 cm^−1^, 1161 cm^−1^, 753 cm^−1^ and 890 cm^−1^, 797 cm^−1^ wavenumbers, respectively.^27^ In the FTIR spectra of Fe_3_O_4_@SiO_2_ NPs and Fe_3_O_4_@SiO_2_@Au a peak at 1560 cm^−1^ corresponding to C–N vibration is visible. The presence of these peaks indicates that the Fe_3_O_4_ NPs were successfully modified by the SiO_2_ groups, thus forming Fe_3_O_4_@SiO_2_ nanoparticles.^28^

**Fig. 5:**
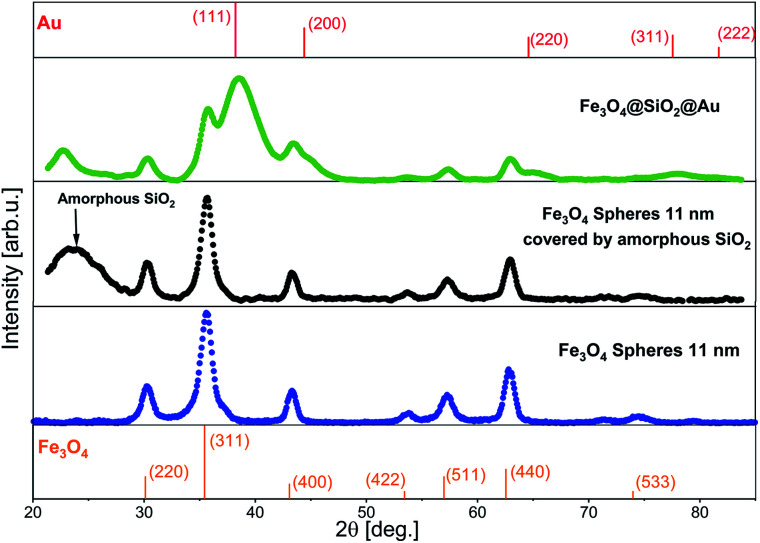
XRD patterns of Fe_3_O_4_, Fe_3_O_4_@SiO_2_ and Fe_3_O_4_@SiO_2_@Au NPs. On the top and on the bottom of the image standard references of Au and of Fe_3_O_4_ are added.^36,37^

The crystalline structure of the NPs fabricated at every stage of the synthesis was investigated using XRD (Fig. 5). The XRD pattern of Fe_3_O_4_ was refined with the Rietveld method using the Fullprof software,^29^ where for peaks fitting a Voigt function in the Thompson-Cox-Hastings approximation was applied.^30^ The prominent peaks at 30.17° (220), 35.54° (311), 43.20° (400), 53.59° (422), 57.13° (511), 62.74° (440) and 74.23° (533) matched the magnetite phase. The calculated lattice constant in the Fullprof program was *a* = 8.361(1) Å. The obtained value is the average value of the lattice constant for bulk magnetite (*a* = 8.39 Å)^31^ and for bulk maghemite (*a* = 8.33 Å),^32^ what is typical for Fe_3_O_4_ NPs.^33^ The XRD pattern of the Fe_3_O_4_@SiO_2_ core–shell NPs looks almost the same as the pattern for pure Fe_3_O_4_ NPs, except the broad peak, which originates from the amorphous SiO_2_. The XRD pattern of the Fe_3_O_4_@SiO_2_@Au NPs contains extra wide peaks, which can be assigned to the (111), (200), (220), (311) and (222) diffraction peaks of small Au NPs with fcc structure.^34^ This allows to suggest that the encapsulation process of Fe_3_O_4_ in the SiO_2_ shell has no influence on the initial structure of the Fe_3_O_4_ NPs, which does also not change during the Au NPs deposition, due to good protection function of the SiO_2_ shell.^35^

**Fig. 6:**
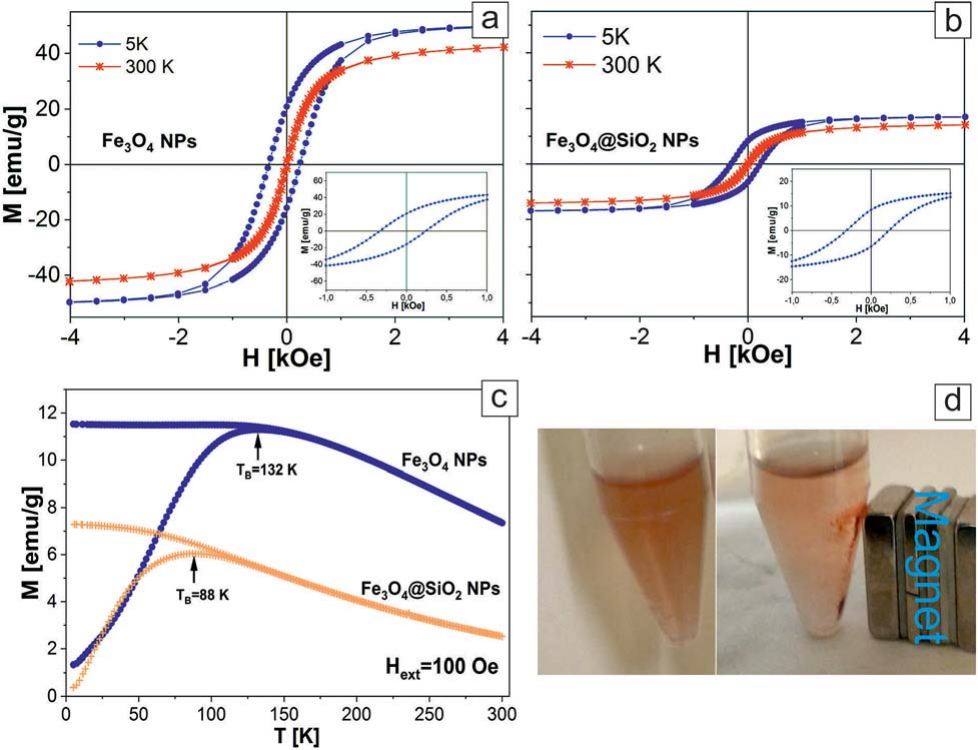
The hysteresis loops of pure Fe_3_O_4_ NPs (a) and Fe_3_O_4_@SiO_2_ NPs (b) measured at 5 K and 300 K. The insets are an expanded view of the low-field region; (c) ZFC-FC curves of pure Fe_3_O_4_ NPs and Fe_3_O_4_@SiO_2_ NPs; (d) Fe_3_O_4_@SiO_2_@Au NPs without magnetic field (left) and attracted by the magnet (right).

In order to ensure that the encapsulation into the SiO_2_ shell has no significant influence on the initial magnetic properties of the Fe_3_O_4_ cores, and to evaluate the application potential of the investigated nanosystems for magnetic resonance imaging, we made magnetometry of the pure Fe_3_O_4_ NPs and the Fe_3_O_4_@SiO_2_ core–shell NPs at room temperature (300 K) and at 5 K (Fig. 6a and b). As seen from the TEM images (Fig. 2), the SiO_2_ shell entirely covers the nanoparticle and protects the iron-oxide core from the environment, preventing its further oxidation. This means that possible changes of magnetic properties of the NPs may occur only at the stage of Fe_3_O_4_ NPs encapsulation into SiO_2_ shell, when the iron-oxide core can be oxidized. Also, as can be seen from Fig. 2b and c, the SiO_2_ shell is the main reason for the relative decrease of iron oxide content in the particles, which will influence the total value of saturation magnetization of the NPs. It was found that at 300 K, pure and core–shell NPs exhibited superparamagnetic behavior with no evident coercivity or remanence.^38^ For Fe_3_O_4_@SiO_2_ NPs saturation magnetization is lower (*M*_S_(300 K) = 15 emu g^−1^) compared to pure NPs (*M*_S_(300 K) = 46 emu g^−1^). The reduced saturation magnetization of the Fe_3_O_4_@SiO_2_ NPs, resulting in decreased magnetic anisotropy and attributed to the relative decrease of iron oxide content in the particles, is mainly explained by the presence of the silica shell coating.^39^ It also worth mentioning that in the case of pure Fe_3_O_4_ NPs, the measured values of *M*_S_ can be reduced by the presence of the protective shell of oleic acid, which prevents the interaction of iron-based NPs with the atmosphere.^33,40^ The temperature dependence of the magnetic moment *M*(*T*) of the Fe_3_O_4_ and Fe_3_O_4_@SiO_2_ NPs under an applied magnetic field (*H*_ext_ = 100 Oe) after zero-field cooling (ZFC) and after field cooling (FC) was measured from 300 K to 5 K (Fig. 6c). This dependence allows to determine the blocking temperature (*T*_B_) of the NPs, being the temperature above which the majority of the particles becomes superparamagnetic, and above which the FC and ZFC magnetization curves coincide. For Fe_3_O_4_@SiO_2_ NPs *T*_B_ = 88 K, while for the pure Fe_3_O_4_ NPs *T*_B_ is higher (*T*_B_ = 132 K). The smaller value of *T*_B_ for the encapsulated Fe_3_O_4_ NPs can be explained by the reduced dipole–dipole interaction between the iron-based cores of the Fe_3_O_4_@SiO_2_ NPs in comparison to pure Fe_3_O_4_ NPs. Likewise, very interesting results were obtained from FC (*H*_ext_ = 50 kOe) magnetization hysteresis loops recorded at 5 K for pure Fe_3_O_4_ and Fe_3_O_4_@SiO_2_ NPs. As expected at this temperature, the hysteresis loops are opened and the NPs are in ferromagnetic state. Also the hysteresis loops have a clear negative shift from the origin along the field axis and evidence the presence of an exchange bias field (*H*_EB_), which originates from the interface exchange coupling between ferro(ferri) (FM)/antiferromagnetic (AFM).^41^ The presence of the exchange bias field may result in higher *T*_B_ values and coercivity fields (*H*_C_), in comparison to particles, where such fields are absent. For pure Fe_3_O_4_ NPs the values *H*_EB_ = 46 Oe, *H*_C_(5 K) = 287 Oe are close to the values *H*_EB_ = 43 Oe, and *H*_C_(5 K) = 268 Oe for Fe_3_O_4_@SiO_2_ NPs (see the insets in Fig. 6a and b). Chalasani *et al.* showed that in Fe_3_O_4_ NPs produced by the thermal decomposition method, the exchange bias fields originate from the presence of trace amounts of wustite (FeO) and thus this higher content of FeO is responsible for the larger exchange bias fields.^42^ The matching of the values *H*_EB_ and *H*_C_ allows to conclude that encapsulation of Fe_3_O_4_ cores by the SiO_2_ shells does not influence the structure of the Fe_3_O_4_ cores. As it can be expected, the attachment of small Au gold NPs does not influence the magnetic properties of the Fe_3_O_4_ NPs and Fe_3_O_4_@SiO_2_@Au NPs can be easily attracted by a magnet (Fig. 6d).

**Fig. 7:**
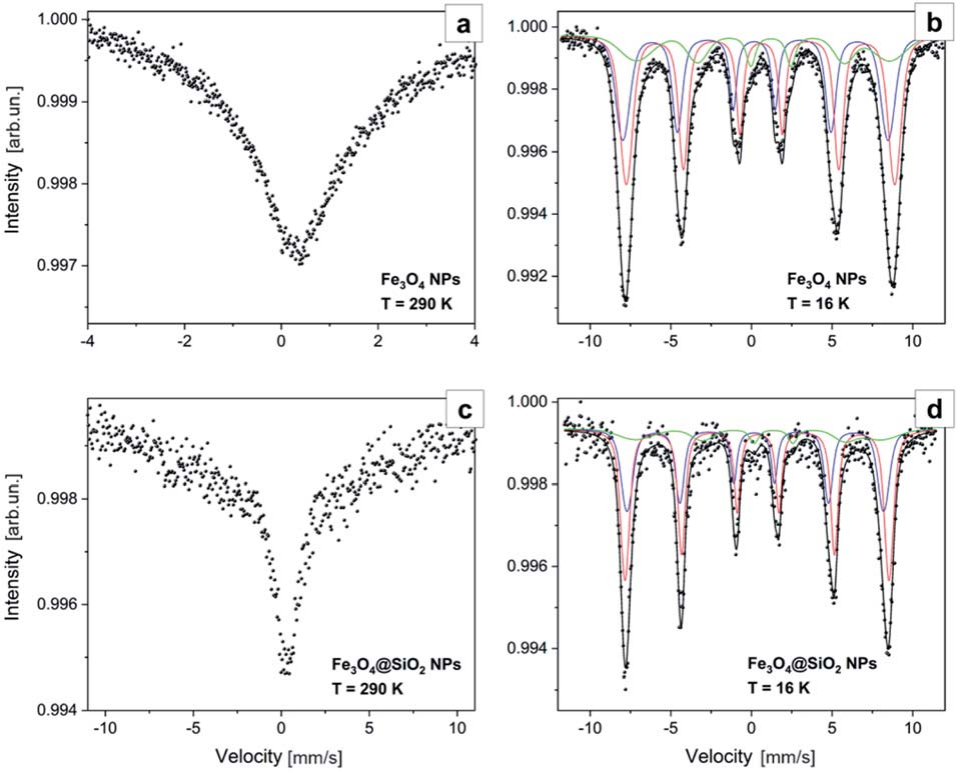
Mössbauer spectra of the Fe_3_O_4_ NPs acquired at 290 K (a) and 16 K (b) and of the Fe_3_O_4_@SiO_2_ NPs acquired at 290 K (c) and 16 K (d).

For local structure characterization of the as fabricated Fe_3_O_4_ cores and after their encapsulation in the SiO_2_ shell, Mössbauer spectroscopy measurements were performed. Fitted ^57^Fe Mössbauer spectra are shown in Fig. 7, while hyperfine parameters of sub-spectra and their relative contribution (area ratio) are summarized in Table 2. It is established by Chlan *et al.*^43^ and Senn *et al.*,^44^ that above the Verwey transition temperature (125 K), stoichiometric magnetite has a cubic *Fd*3̄*m* symmetry with inverse spinel cubic structure. The cell unit (Fe^3+^)_A_ (Fe^3+^Fe^2+^)_B_ consists of Fe^3+^ ions at the A-site (tetrahedral) and Fe^2+^ ions at the B-site (octahedral). At tetrahedral sites, Fe atoms are in a +3 state, while in B positions the mean valence is +2.5. Mössbauer spectra of the as fabricated Fe_3_O_4_ core samples and after their encapsulation in the SiO_2_ shell at room temperature (Fig. 7a and c), are characterized with singlet IS = 0.32 mm s^−1^, without resolved quadrupole splitting corresponding to Fe^3+^ ions. The observed broadened baseline in spectrum of NPs, is most probably caused by the presence of an unresolved magnetic sextet with weighted average isomer shift ∼0.6 mm s^−1^ corresponding to Fe^2.5+^ ions, which indicates a superparamagnetic state. At low temperatures, the structure of magnetite is monoclinic with a *Cc* space group symmetry.^44^ After Verwey transition, the unit cell becomes four times bigger than cubic, doubled in the *c* direction, and with Fe ions residing in 16 B inequivalent positions (8 Fe^3+^ and 8 Fe^2+^) and 8 A inequivalent positions.^43^ The spectrum of Fe_3_O_4_ NPs (Fig. 7b) obtained at 16 K, is deconvoluted into three sextets with parameters corresponding to Fe^3+^ ions at A site (IS = 0.21 mm s^−1^ and *H*_hf_ = 51.1 T), Fe^3+^ ions at B site (IS = 0.58 mm s^−1^ and *H*_hf_ = 51.7 T) and Fe^2+^ ions at B site (IS = 0.96 mm s^−1^ and *H*_hf_ = 48.8 T), which is in good agreement with the values presented in ref. 43. It is worth mentioning, that according to the results of spectra approximation (Table 2), the ratio of the contribution of sextet of Fe^3+^ ions at A site to the contribution of sextets of Fe ions at B site is 0.56, which is very close to the reference value of stoichiometric magnetite.^45,46^ At the same time the relative contribution of subspectrum attributed to Fe^2+^ ions at B site is less than expected (only 17% against ∼30% expected). The lower contribution of Fe^2+^ ions in Mössbauer spectrum could be due to the partial oxidation of Fe_3_O_4_ NPs leading to the formation of some amount of maghemite or wustite at the surface of NPs. The Mössbauer spectra of the Fe_3_O_4_ cores after their encapsulation in the SiO_2_ shell (Fig. 7d) are very similar to those of the Fe_3_O_4_ NPs, but with slightly smaller values of IS and *H*_hf_. As it was mentioned by Lyubutin *et al.*,^47^ the shell material can induce pressure on the NPs and this effect can change the Mössbauer parameters. From the other side, the shift of IS and *H*_hf_ values may be attributed to the formation of interfacial structure.^48^ The above-mentioned results confirm that the as-fabricated Fe_3_O_4_ NPs and those encapsulated by the SiO_2_ shell possess a very similar crystal structure.

**Table 2:** Fitting parameters of Mössbauer spectra approximation

Sample (temperature)	Sub-spectrum	IS, mm s^−1^	*H*_hf_, T	*H*_hf_-sigma, T	*E*, mm s^−1^	Area ratio, %
Fe_3_O_4_ NPs (290 K)	Fe^3+^/Fe^2.5+^	0.32	—	—	—	26
Fe_3_O_4_ NPs (16 K)	Fe^3+^ at A site	0.21	51.1	0.14	0.04	36
	Fe^3+^ at B site	0.58	51.7	0.12	−0.02	47
	Fe^2+^ at B site	0.96	48.8	5.5	−0.24	17
Fe_3_O_4_@SiO_2_ NPs (290 K)	Fe^3+^/Fe^2.5+^	0.32	—	—	—	26
Fe_3_O_4_@SiO_2_ NPs (16 K)	Fe^3+^ at A site	0.20	49.4	0.11	0.04	35
	Fe^3+^ at B site	0.38	50.8	0.07	−0.04	53
	Fe^2+^ at B site	0.90	47.2	6.1	−0.47	12

### MR imaging of Fe_3_O_4_@SiO_2_

**Fig. 8:**
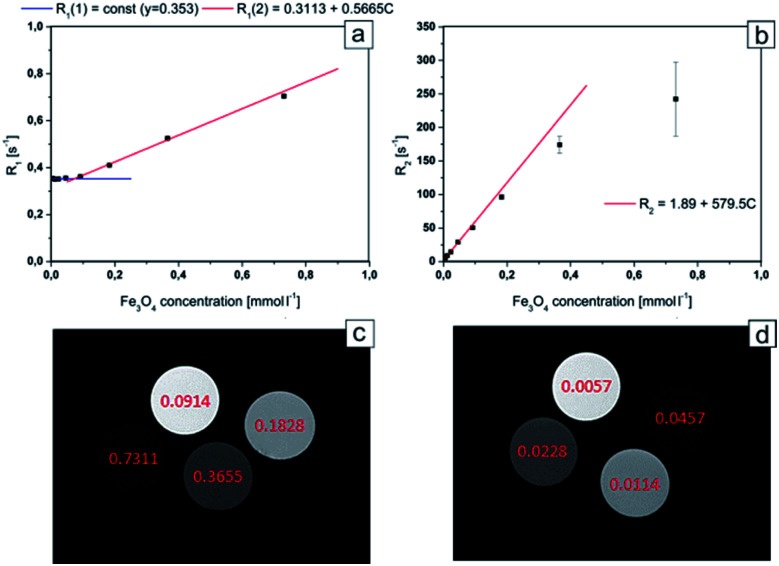
*R*_1_ relaxation rate dependence on Fe_3_O_4_ concentration for Fe_3_O_4_@SiO_2_ nanoparticles (a); *R*_2_ relaxation rate dependence on Fe_3_O_4_ concentration for Fe_3_O_4_@SiO_2_ nanoparticles (b); axial images of samples containing different concentration of Fe_3_O_4_@SiO_2_ nanoparticles. MSME sequence: *T*_R_: 6000 ms, *T*_E_: 8 ms, FA: 180°, FOV: 40 × 40 mm, slice thickness: 2 mm; for *T*_E_ = 8 ms (c) and 105 ms (d) respectively. Concentrations of Fe_3_O_4_ are marked in pictures (in mmol L^−1^).

In order to evaluate the theranostic potential of the studied nanoparticles, we investigated the MRI enhancing function of the active element of the nanoparticle (*i.e.* Fe_3_O_4_@SiO_2_), which can influence the MRI contrast. In this experiment we investigate only the active element of the nanoparticle because it was the only component exhibiting strong magnetic properties, causing local perturbations of the magnetic field, which resulted in signal loss due to significant shortening of the transversal relaxation time *T*_2_. In addition, neither Au nanoparticles (see Fig. 2g) nor MHDA linker (see Fig. 1) form a continuous shell around the Fe_3_O_4_@SiO_2_ NPs. As a result, the Au NPs and attached drugs did not interfere to prevent water molecules from possible direct contact with the surface of the active element. Alternative behavior was observed for the case of continuous shell observed for NPs studied, for example, by Hou *et al.*^14^ where Fe_3_O_4_@SiO_2_ NPs were covered by 15 nm gold continuous shell. In our case the water molecules were very close to the surface of Fe_3_O_4_@SiO_2_ NPs and the dephasing of protons was activated by magnetic field perturbations in the direct vicinity of the Fe_3_O_4_@SiO_2_ NPs. Thus, for MRI contrasting properties evaluation, we calculated the values of the relaxation rates: *R*_1_ and *R*_2_ from the measured relaxation times for Fe_3_O_4_@SiO_2_ active element of the NPs. The dependences of the relaxation rates *R*_1_ and *R*_2_ on the concentration of Fe_3_O_4_@SiO_2_ nanoparticles are presented in Fig. 8a and b respectively. The effect of increasing NPs concentration on the relaxation rate is significantly higher for *R*_2_, especially in low concentration range, most interesting for potential clinical application of the theranostic agents. In a concentration range up to 0.1828 mmol L^−1^, there was a very good linear dependence of nanoparticles concentration on the *R*_2_ relaxation rate (*R*_2_ above 0.99). For higher concentrations, the effect of *T*_2_ shortening was less pronounced, which can be attributed to clustering of NPs, as their concentration in the solution increases.^49^ This effect of NPs clustering was observed visually in samples with the highest concentration (Fe_3_O_4_ concentrations: 0.7311 and 0.3655 mmol L^−1^), therefore the specific relaxivity *r*_2_ was determined only for unclustered NPs. Linear regression results for *R*_2_ relaxation rates were: *R*_2S_ = 1.894 ± 0.033 s^−1^, *r*_2_ = 579.5 ± 4.3 s^−1^ mmol^−1^ L. Conversely, the effect of *T*_1_ shortening was practically not observed for low NPs concentrations up to 0.0457 mmol L^−1^, with *T*_1_ values characteristic for distilled water, and in higher concentration range, it was much weaker than observed for *T*_2_. This effect was confirmed by other papers^5,14^ and can be attributed to limited access of water molecules to superparamagnetic center (caused by SiO_2_ coating), which is crucial for *r*_1_ enhancement. Only significantly higher concentrations of Fe_3_O_4_@SiO_2_ NPs, provide enough superparamagnetic centers to effectively change the *T*_1_ relaxation time of the whole sample.^50^ Linear regression results for *R*_1_ relaxation rate were: *R*_1S_ = 0.3113 ± 0.0020 s^−1^, *r*_1_ = 0.5665 ± 0.0070 s^−1^ mmol^−1^ L, illustrating three orders of magnitude difference between *r*_2_ and *r*_1_ relaxivities.

The obtained results showed that the examined nanocapsules exhibit a strong effect of *R*_2_ relaxation rates. In lower concentration range, which is more interesting in view of possible clinical applications, the effect of *T*_1_ relaxation time is very small, and will have negligible effect on the MR image contrast change. Therefore, the investigated Fe_3_O_4_@SiO_2_ nanoparticles, exhibit very strong *T*_2_ contrasting properties (“negative” contrast), which is greatly visible in *T*_2_-weighted images (see Fig. 8c and d). Here, the MR images for high and low NPs concentration ranges are compared separately, to illustrate the possibility of effective contrasting in both cases. For high concentrations however, much shorter echo time (*T*_E_) in MRI pulse sequences have to be applied to get reasonable images.

### Stability of the biofunctionalized Fe_3_O_4_@SiO_2_@Au NPs with immobilized cPt on their surface

In order to evaluate the stability of the functionalized Fe_3_O_4_@SiO_2_@Au NPs and to verify the effectiveness of the biofunctionalization and immobilization process, Raman spectroscopy, as well as TGA and DSC analysis, have been performed.

**Fig. 9:**
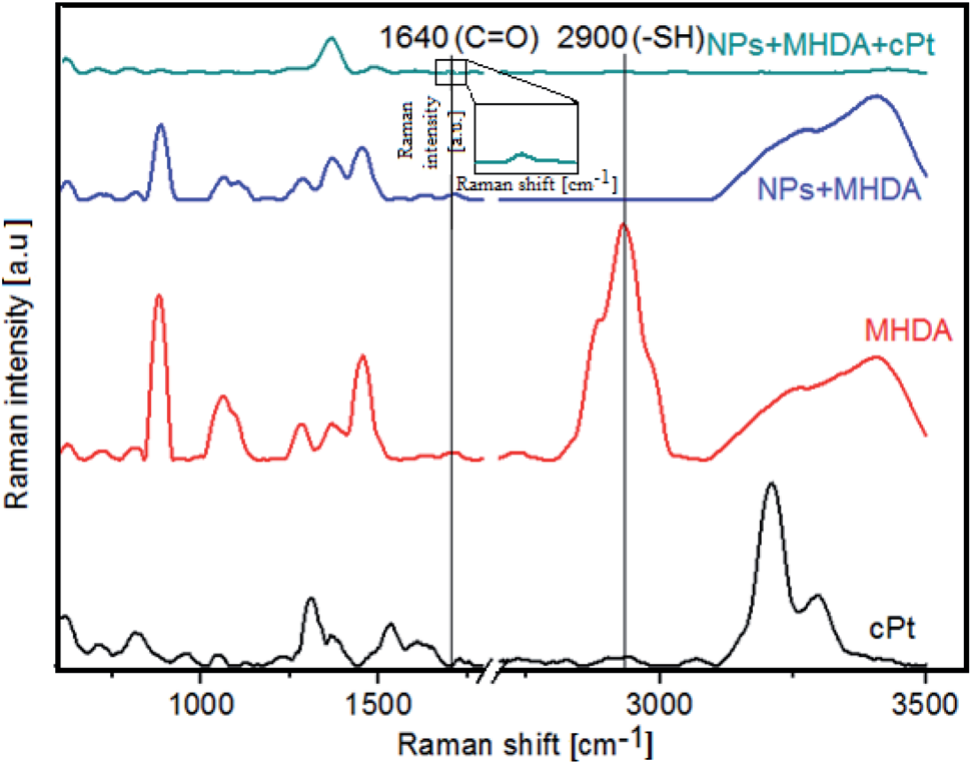
Raman spectra of: cPt (black spectrum), the pure MHDA (red spectrum), the Fe_3_O_4_@SiO_2_@Au + MHDA (blue spectrum), Fe_3_O_4_@SiO_2_@Au + MHDA + cPt (green spectrum).

In this study, we show a successful biofunctionalization of Fe_3_O_4_@SiO_2_@Au, which is very important for an effective delivery of the drug to cancer cells. For this purpose, Raman spectra of four samples *i.e.* cPt (Fig. 9, black spectrum), pure MHDA (Fig. 9, red spectrum), the MHDA capped Fe_3_O_4_@SiO_2_@Au NPs (Fig. 9, blue spectrum) and Fe_3_O_4_@SiO_2_@Au NPS + MHDA + cPt (Fig. 9, green spectrum) were acquired. In the Raman spectrum of the MHDA sample a peak of the thiol group (–SH) at 2900 cm^−1^ is visible.^51^ The disappearance of this peak in the biofunctionalized and cPt containing samples confirms a successful attachment of the MHDA to the surface of gold NPs. Moreover, in the Raman spectrum of Fe_3_O_4_@SiO_2_@Au + MHDA + cPt (Fig. 9, green spectrum), a peak corresponding to the C=O vibrations (1680 cm^−1^) is observed.^52^ This group is responsible for linking cPt with biofunctional surfactants on the Fe_3_O_4_@SiO_2_@Au surface.^51^ Both observations provide an evidence on the success of Fe_3_O_4_@SiO_2_@Au biofunctionalization and cPt immobilization.

**Fig. 10:**
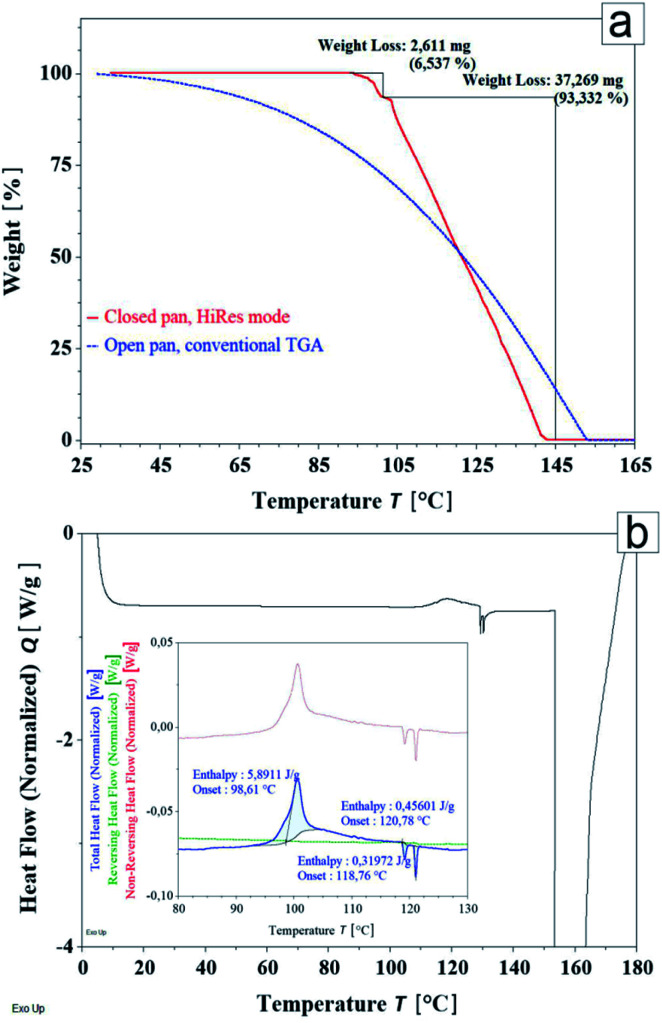
Conventional (dashed line) and HiRes mode (solid line) TGA (a) and DSC (b) experiments performed for functionalized Fe_3_O_4_@SiO_2_@Au NPs. The inset presents components of the heat flow signal registered in MT DSC experiment.

The results of TGA experiments presented in Fig. 10 a, were measured for liquid functionalized Fe_3_O_4_@SiO_2_@Au NPs and were acquired either directly in an open pan or using a hermetic aluminium container with a hermetic lid, automatically punctured right before loading the sample into the TGA furnace. While the curve registered in the conventional TGA mode shows only strong evaporation of the solvent, the data recorded during the HiRes mode experiment (solid line), indicate two major weight loss regions. First, a small, but still pronounced decrease of mass starts slightly above 93 °C (7.14% weight loss), and the second, much larger one, follows from *ca.* 103 °C (92.8% weight loss). Most probably both observed effects are due to the conversion of the solvent itself, rather than of the active pharmaceutical ingredient.

The DSC thermogram registered upon heating is dominated by a broad peak starting around 147 °C, originating from an endothermic process reflecting a decomposition of the sample. Nevertheless, a group of small thermal anomalies is also visible between 95 °C and 122 °C (Fig. 10b). A closer inspection of the heat flow curve measured during the MTDSC experiment (inset in Fig. 10b), revealed one exothermic thermal effect followed by two endothermic ones. Further analysis of the heat flow signal allowed to conclude that these anomalies are strictly kinetic, since they are contributing to the non-reversing (kinetic) component of the measured heat flow.

The results of both TGA and DSC measurements, indicate that the specimen is thermally stable at least up to 90 °C. However, further study of the anomalies revealed during DSC and TGA experiments above 90 °C are necessary and might shed new light on decomposition kinetics of the functionalized Fe_3_O_4_@SiO_2_@Au NPs.

### Simulation of combined chemo-phototherapy of colon cancer cells

#### Morphology of colon cancer cells

**Fig. 11:**
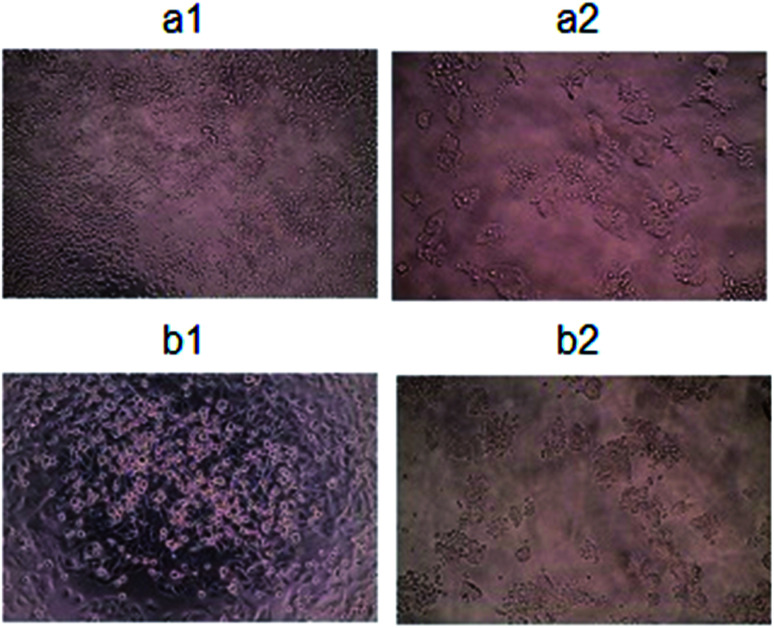
Microscopy images of colon cancer cells (SW480, SW620) morphology: Ctrl (a1 and b1); C@808cPtNPs (a2 and b2, respectively).

The morphological analysis of the two lines of colon cancer cells (Fig. 11), provides information about the influence of functionalized Fe_3_O_4_@SiO_2_@Au NPs and laser irradiation effects on the cells. Detailed information about the morphology changes in cells cultured with functionalized and non-functionalized NPs, with cPt, with and without irradiation are presented in the ESI in Fig. S1.† The changes of the morphology of cells were compared with the control SW480 and SW620 cell lines presented in Fig. 11a1 and b1, respectively. Light microscopy images of SW480 and SW620 cells cultured with Fe_3_O_4_@SiO_2_@Au + cPt and irradiated with an 808 nm laser, showed the largest changes in the morphology and the smallest number of living cells (Fig. 11a2 and b2). In comparison with the control, only small differences in the morphology of cells from both cell lines were visible in the microscopy images of cells cultured with non-functionalized nanoparticles (Fig. S1a1 and b1†). These cells had a spherical shape and evidently adhered to the substrate. Strikingly, the addition of functionalized nanoparticles to the cells and their subsequent irradiation, showed a strong effect on both: cell morphology and cell death. It was related to the immobilization of cPt on the surface of NPs and the presence of the small Au NPs at the surface of the Fe_3_O_4_@SiO_2_ NPs. Cisplatin involves creating a cross-linking between adjacent DNA strands and within the same strand. The formation of these crosslinks prevents DNA replication and cell division.^53,54^ Moreover, the presence of Pt in the structure of cPt causes, that this drug has catalytic properties, which are better at high temperatures.^55^ The high temperature is induced by the laser irradiation of the 4 nm Au NPs, which absorb light and subsequently they convert the light energy into heat energy. This conversion is caused by several photo-physical processes, which take place one after the other.^56^ Firstly, light absorbed by Au NPs is quickly converted to heat and forms a hot metallic lattice by two processes: electron–electron relaxation, which is observed in femtoseconds and electron–phonon relaxation – picoseconds. During these processes, the temperature in the Au NPs could increase to about 1000 K. When the light beam is turned off, the phonon–phonon relaxation is stopped and the heat energy goes to the cells.^57^ The cancer cells are sensitive to high temperatures, therefore, the laser irradiation combined with Au NPs causes death of cells, which was observed in the Fig. 11a2 and b2 as cells were not sticking to the ground. Moreover, the small size of Au NPs caused, that 100% of the light energy was converted into heat energy.^58^

#### Viability

**Fig. 12:**
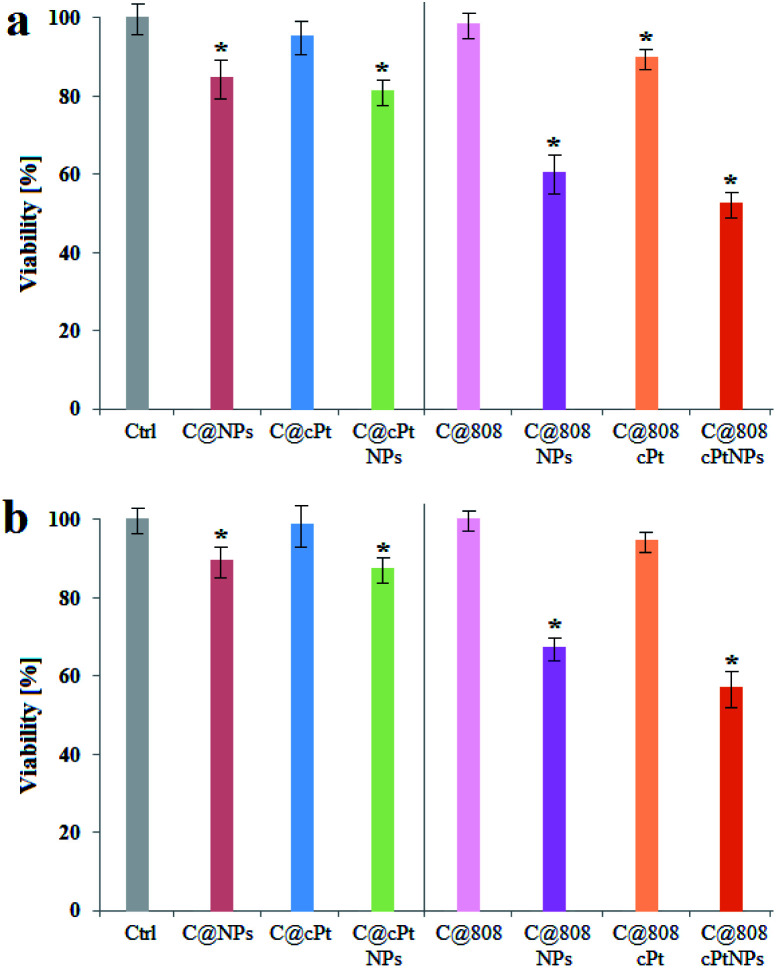
Viability of colon cancer cells: SW480 (a); SW620 (b) after addition of cPt, Fe_3_O_4_@SiO_2_@Au, functionalized Fe_3_O_4_@SiO_2_@Au and laser irradiation in the presence of these three systems. Data was considered as significant when *p < 0.05 *vs.* control.

The viability test showed, that the smallest cytotoxic effect for both SW480 and SW620 cell lines was observed for the C@808, ∼2% and C@cPt, 4% and 2% samples. Moreover, the MTS assay showed a relatively small decrease of viability of cancer cells cultured with the Fe_3_O_4_@SiO_2_@Au NPs. The small cytotoxicity of Fe_3_O_4_@SiO_2_@Au NPs can be caused by the medium, in which the cells were cultured, because this medium reduces the repulsive forces among the NPs, therefore aggregation of Fe_3_O_4_@SiO_2_@Au NPs, was possible.^59^ However, a certain toxicity effect was observed in the MTS results, Fig. 12. It can be caused by the interaction between the nanoparticles and the proteins, which are in the cell membranes.^60^ Furthermore, the accumulation of nanoparticles, as well as their cytotoxicity effect is dependent not only from their size or shape, but also from cells line, which was cultured with the nanoparticles.^61^ Therefore, in our study we observed differences in the viability between the cells from different lines. Moreover, the observed toxicity differences between the cell lines, could be caused by the fact that the SW480 cell line originates from the primary tumor, whereas the SW620 line is from a metastatic lesion to the lymph node, although both cell lines originate from the same patient. The metastatic lesion cells are very often more aggressive than cells from the primary tumor.^62^

Laser irradiation of the cells cultured with cPt caused 12% and 8% mortality of the SW480 and SW620 cell lines, respectively, which is around three and four times higher, than the mortality effect of cells cultured with cPt, but not subjected to irradiation.

Cells cultured with non-functionalized Fe_3_O_4_@SiO_2_@Au NPs and irradiated by laser, exhibited a very high mortality in comparison with other samples. This effect was caused by the Au NPs decorating the Fe_3_O_4_@SiO_2_ NPs. Gold NPs show photothermal effects of plasmon-resonance, which depend, among others, on the NPs size. Therefore, it can be assumed, that Au NPs were used as photosensitizers in this study. The temperature effects, correlated with the nanoparticle sizes were theoretically explained by Jain *et al.*^63^ They showed the relationship existing between the size of the Au NPs diameter and the optical absorption or scattering properties of these nanoparticles using the Mie theory. Indeed, for the gold nanoparticles with ∼20 nm diameter, the main contribution to the total extinction of light was absorption. Interestingly, with the increase of the nanoparticle size up to ∼40 nm, the values of light extinction were dependent on the scattering effects. When the particle size was ∼80 nm, the extinction of gold nanoparticles was caused by both, absorption and scattering.^64^ Moreover, Jiang *et al.* calculated, using experimental data as well as the Mie theory, the dependence between the particle size and photothermal conversion efficiency of spherical gold nanoparticles and they showed, that smaller nanoparticles can more efficiently transduce the energy from light to heat than larger Au NPs.^65^ The highest, ∼50% and ∼43% mortality of the SW480 and SW620 cells were observed in the case of C@808cPtNPs samples. This could be caused by synergistic effect of both components cPt and Au NPs, as well as the enhancement by laser irradiation.^66^

## Conclusions

In conclusion, a new type of compact Fe_3_O_4_@SiO_2_@Au nanoparticles (below 40 nm in size) for theranostic applications was designed, synthesized and investigated. The fabricated nanoparticles were uniform, well dispersed in water and had low distribution in sizes of the spherical Fe_3_O_4_ core (≈11 nm), the thickness of SiO_2_ shell (≈10 nm), and Au nanoparticles (≈4 nm) deposited on SiO_2_ shell. The investigated nanoparticles showed pronounced superparamagnetic properties at room temperature with high saturation magnetization, which is caused by Fe_3_O_4_ core. The superparamagnetic state was confirmed by Mössbauer spectroscopy, which showed a short relaxation time of the NPs magnetic cores. It was found that the structure and magnetic properties of the Fe_3_O_4_ core were stable and were very similar before and after their encapsulation in the SiO_2_ shell, which stabilized them and made dispersible in water. The studied nanoparticles exhibited very strong *T*_2_ contrasting properties, which is nicely visible in *T*_2_-weighted images, and which made the nanoparticles applicable in diagnostics, as contrast agents in MRI. The investigated nanoparticles also demonstrate low toxicity, because of the biocompatible SiO_2_ continuous shell, which reliably prevents the contact of the Fe_3_O_4_ magnetic core with the environment. Moreover, functionalized nanoparticles showed a synergetic effect of investigated nanoparticles and anticancer drugs in chemo-photothermal stimulated treatment of cancer. All of the as mentioned properties of the nanoparticles showed their potential in magnetic resonance imaging (MRI)-guided chemo-photothermal stimulated treatment of cancer.

## Conflicts of interest

There are no conflicts to declare.

## Supplementary Material

RA-010-D0RA03699D-s001
