# Supplementary material for: Fe3O4@SiO2@Au nanoparticles for MRI-guided chemo/NIR photothermal therapy of cancer cells
Source: RSC Adv. 2020 Jul 15;10(44):26508–20. doi: 10.1039/d0ra03699d (PMC9055504; doi:10.1039/d0ra03699d)
Supplement: RA-010-D0RA03699D-s001 [file RA-010-D0RA03699D-s001.pdf]

Electronic Supplementary Material (ESI) for RSC Advances.
This journal is © The Royal Society of Chemistry 2020

Supplementary materials

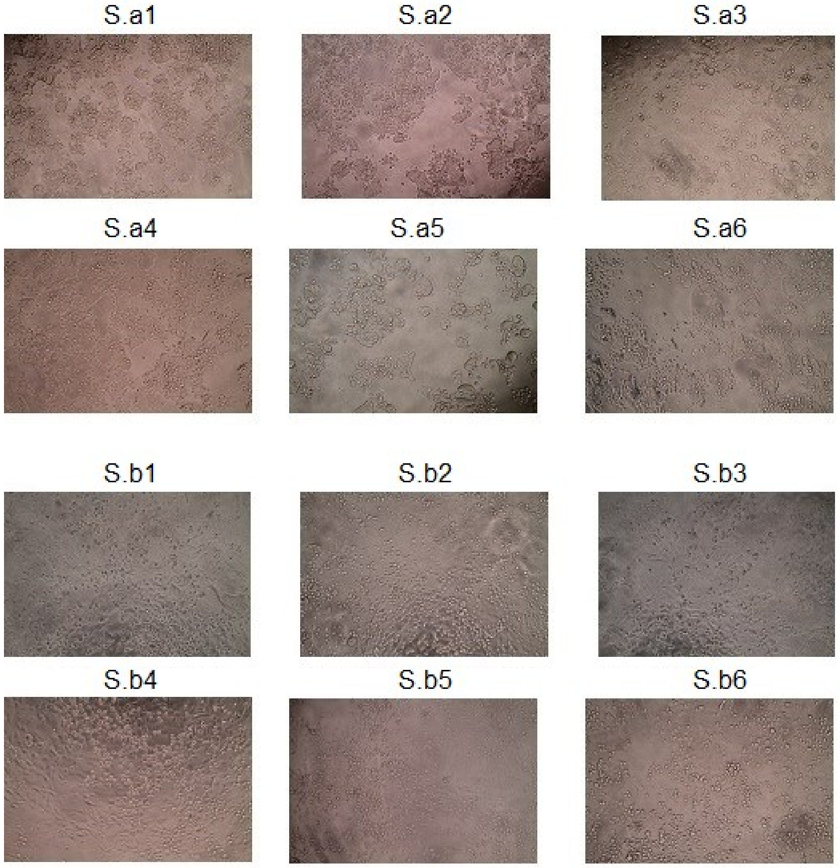

Figure S1. Microscopy images of colon cancer cells (SW480, SW620) morphology: C@NPs (S.a1, S.b1, respectively); C@cPt (S.a2, S.b2, respectively); C@cPtNPs (S.a3, S.b3, respectively); C@808 (S.a4, S.b4, respectively); C@808NPs (S.a5, S.b5, respectively); C@808cPt (S.a6, S.b6, respectively).
